# Self-management in face-to-face peer support for adults with type 2 diabetes living in low- or middle-income countries: a systematic review

**DOI:** 10.1186/s12889-020-09954-1

**Published:** 2020-11-30

**Authors:** Melanie Pienaar, Marianne Reid

**Affiliations:** University of Free State, 205 Nelson Mandela Drive, Park West, Bloemfontein, 9301 South Africa

**Keywords:** Type 2 diabetes, Face-to-face, Peer support, Self-management, Low-and middle-income countries

## Abstract

**Background:**

Peer support has been recognised as a promising strategy to improve self-management in patients living with chronic conditions, such as Type 2 diabetes (T2D). The purpose of the review was to synthesise the best available evidence on face-to-face peer support models for adults with T2D in low and middle-income countries (LMICs).

**Methods:**

We searched Medline, Cumulative Index to Nursing and Allied Health, Literature Academic Search Ultimate, PsycINFO, CAB Abstracts, Health Source: Nursing/Academic Edition, SPORTDiscus, Africa-Wide Information, MasterFILE Premier, SocINDEX, ERIC, PsycARTICLES, Open Dissertations, Communication & Mass Media Complete, Health Source-Consumer Edition and Google Scholar for the period January 2000 to December 2017. Reference list checking and contact with authors were additional sources of data. Screening of papers, critical appraisal and data extraction were carried out independently by at least two reviewers.

**Results:**

From 3092 abstracts retrieved from database searches, data was extracted from 12 papers. There was no consistency in design, setting, outcomes or measurement instruments amongst the papers. The papers were associated with improvements in various clinical and behavioural outcomes. Diabetic patients and community health workers (CHWs) were identified as two common face-to-face peer support models. The recruitment and selection of diabetic patients as peer supporters focused on patients from the community, with good glycaemic control and/or leadership skills, who were recommended by healthcare professionals. Recruitment of CHWs as peer supporters was done from an existing infrastructure of CHWs in the community and, thus, selection criteria were poorly described. The training of peer supporters featured as an important component, highlighting who provided training and the duration and content covered in training. Motivational interviewing was the most common theory basis of training used in the peer support interventions. Face-to-face, group and/or individual-based peer support was often supplemented by other peer support methods. The supervision of peer supporters was generally poorly described.

**Conclusions:**

The comprehensive synthesis of the best available evidence has led to new insights regarding face-to-face peer support as a self-management strategy for patients with T2D in LMICs. Face-to-face peer support may be implemented in innovative ways to improve the quality of life of patients with T2D.

**Trial registration:**

PROSPERO trial registry number, CRD 42018103261.

**Supplementary Information:**

The online version contains supplementary material available at 10.1186/s12889-020-09954-1.

## Background

Diabetes is causing a global health crisis. Since 2000 until today, the global prevalence of diabetes type 1 and 2 combined, has grown exponentially from 151 million to 463 million. Approximately half a billion people are presently living with diabetes and 80% of the diabetes burden is carried by low and middle-income countries (LMICs) [1]. In this complex chronic disease, the patient is responsible for more than 95% of the management of diabetes [2, 3]. Self-management by people with type 2 diabetes (T2D) implies following a healthy diet, incorporating physical exercise into the daily routine, using medication correctly; monitoring blood glucose levels, recognising and responding to warning signs, and making the right decisions regarding healthcare [4–6]. These requirements can present a daunting situation for the patient, especially if the patient lacks knowledge, resources and social support. Many patients cannot manage their condition without continuous support. In this respect, peer support has been identified as a promising strategy to improve diabetes self-management [7–9].

Peer support can be described as support from an individual who shares similar characteristics or experiences as the patient [10–12]. The uniqueness of peer support is that peer supporters, as defined above, may understand the languages, cultures and circumstances of the patients they serve, thereby creating an immediate connection with patients, and making it easier to share challenges and experiences with an insider. Peers for Progress, a global evidence-based initiative, identified the following functions of peer support namely assistance in daily care; social and emotional support; linkage to care and ongoing support [13]. Literature describes different peer support models for T2D, such as models led by healthcare professionals, diabetic patients and/or community health workers (CHWs). Furthermore, these models may be delivered in various modes such as face-to-face, telephoned-based and/or internet-based [13].

This study’s specific focus on face-to-face peer support models was informed by a study on the development of a health dialogue model for patients with diabetes in LMICs [14]. During this model development, the findings emphasised three areas: the need to improve community awareness regarding diabetes; the use of face-to-face communication, such as peer support, or technological support such as mobile health devices, to promote the self-management of T2D in patients and creating a diabetes training platform for healthcare providers caring for patients with T2D [14].

Several systematic reviews have demonstrated the impact of peer support on diabetes and self-management outcomes, such as on glycosylated haemoglobin (HbA1c), body mass index, self-efficacy, physical activity and blood pressure [15–20]. However, there is no systematic review, as far as the authors could determine, on any face-to-face – either group or individual-based – peer support models for adults with T2D in LMICs. To bridge this gap in the existing literature, this systematic review will be used to determine which face-to-face peer support model(s) is identified as a self-management strategy for patients with T2D in LMICs? Furthermore, this systematic review will be used to inform the development and implementation of the identified face-to-face peer support intervention in a pilot within a complex intervention in a LMIC. The questions that were addressed in this review are: 1) What face-to-face peer support models were identified as a self-management strategy for patients with T2D in LMICs and 2) how were they designed and implemented?

### Theoretical grounding

**Fig. 1:**
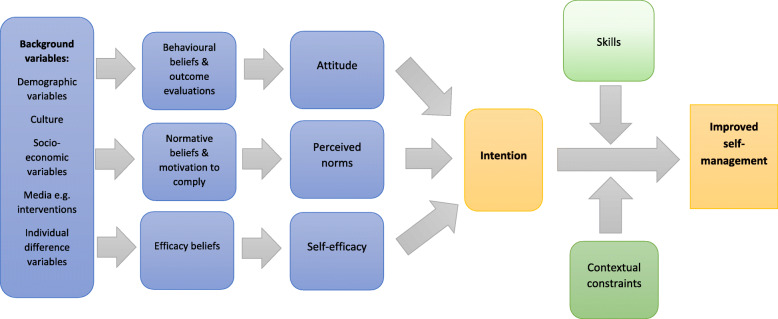
Integrated Model of Behaviour Prediction (adapted from [23])

The theoretical grounding of the study was provided by the integrative model of behavioural prediction (IMBP) due to its potential for behaviour change [21]. The IMBP is an extension of the theory of reasoned action and the theory of planned behaviour [22]. The IMBP “*extends the scope of the normative determinant and points attention to skills and environmental barriers as moderators of the intention–behaviour relationship*” [21]. The IMBP, as depicted in Fig. 1, suggests that an individual will perform the intended behaviour if the necessary skills are present and if contextual factors allow the behaviour to be performed. However, if the individual does not carry out the behaviour, it is generally due to lack of skills and/or contextual factors, and not due to lack of motivation. Background factors such as demographics, culture, socio-economic situation, exposure to media and individual factors such as personality and personal experience are sources of belief. These beliefs may influence attitude towards behaviour, perceived norms and self-efficacy and eventually intention to perform behaviour [23]. In this study, a systematic review was performed, as face-to-face peer support could serve as a strategy to develop self-management skills and minimise contextual constraints.

## Methods

This study is registered with PROSPERO, trial registry number CRD 41018103161, and was conducted according to the Preferred Reporting Items for Systematic Reviews and Meta-Analyses (PRISMA) guidelines [24].

### Data sources and searches

A librarian experienced in systematic reviews assisted with the search strategy. The following electronic databases were searched: Medline, Cumulative Index to Nursing and Allied Health, Literature Academic Search Ultimate, PsycINFO, CAB Abstracts, Health Source: Nursing/Academic Edition, SPORTDiscus, Africa-Wide Information, MasterFILE Premier, SocINDEX, ERIC, PsycARTICLES, OpenDissertations, Communication & Mass Media Complete, Health Source-Consumer Edition and Google Scholar based on these databases’ accessibility and comprehensiveness. The data searches were conducted from 1 January 2000 to 30 December 2017, a period where diabetes prevalence escalated. Additionally, the reference lists of studies included were reviewed for relevance, and authors of studies were contacted if additional information was required. The search terms used in the electronic search strategy are provided in Table 1.

**Table 1:** Search terms used in the electronic search

Variables	Search words
**Population:** Adults patients with T2DM	(“diabetes mellitus*” or “type 2 diabet*” or “type 2 diabet*” or diabetic or niddm or “non-insulin dependent diabet*” or iddm or “insulin dependent diabet*” or t2d or “t2 dm”)
**Intervention:** Peer support intervention	(“lay worker*” or “lay health worker*” or volunteer* or coach* or “patient* navigator*” or “community health worker*” or “health advisor*” or promotora* or “outreach worker*” or “health representative*” or “lay health educator*” or peer*) and (interven* or educat* or counsel* or support*)
**Comparison:** Standard care	usual care
**Outcome:** Improved self-management	(“self manag*” or “health behav*” or hba1c or weight* or “blood pressure” or “health litera*” or “self-care*” or “self caring” or “self effic*”)

### Study selection

Two independent reviewers (MP and MR) screened the titles and abstracts of potential papers against the following *inclusion criteria*: papers in English, or in other languages with an English abstract; papers reporting on studies that had been conducted in LMICs; papers of studies involving adults (age ≥ 18) diagnosed with T2D by physicians; and papers reporting group or individual face-to-face peer support. All study designs were included. *Papers were excluded* if participants were <18 years of age; if papers were in languages other than English and had no English abstract; and if papers reflected studies that were conducted in high-income countries. Furthermore, papers of peer support interventions led by professional healthcare workers, as well as telephone, web and internet-based peer support interventions as primary mode of delivery, were excluded. Papers in the form of editorials, research briefs and conference reports were also excluded. Inclusion of the papers was guided by the World Bank’s classification criteria for countries in LMICs that defined low-income economies as having a gross national income (GNI) of $1025 or less; lower middle- income economies having a GNI between $1026 and $3995 and upper middle-income economies having a GNI between $3996 and $12,375 [25]. These countries may have limited access to resources, trained specialists, infrastructure and technology [26]. Two independent reviewers (MP and MR) assessed the full texts of potential papers that were managed in Mendeley, an online reference manager.

### Data extraction and quality assessment

One reviewer (MP) extracted data from the potential papers using a standardised data extraction form. The information that was captured included bibliographic details and context, methodology, intervention and outcomes, study findings and quality assessment rating. A second reviewer (MR) then verified the accuracy of the data. The following reviewers (MP, MR, EJvR and CS) carried out quality assessment of the potential studies. The following tools were used to assess the risk of bias of the papers according to their study design: Critical Appraisal Skills Programme (CASP) randomised controlled trial tool [27], CASP review tool [28], and the Johanna Biggs Institute appraisal checklist for quasi-experimental papers [29]. Although the scores differed according to the research design used, the tools focused on the following aspects related to methodological quality: a focused area, process of recruitment, data collection methods, what were the results and could the results be applied to the local population. The quality assessment revealed good, moderate and poor quality papers, with a good quality paper indicating low risk of bias. When discrepancies arose amongst reviewers, consensus was reached through discussion.

### Data synthesis and analysis

The purpose of the review was to synthesise the best available evidence on face-to-face peer support models for adults with T2D in LMICs. Due to the heterogeneity of the studies in terms of design, setting, outcomes and measurement instruments, a meta-analysis was not possible, and a narrative synthesis was performed instead.

## Results

**Fig. 2:**
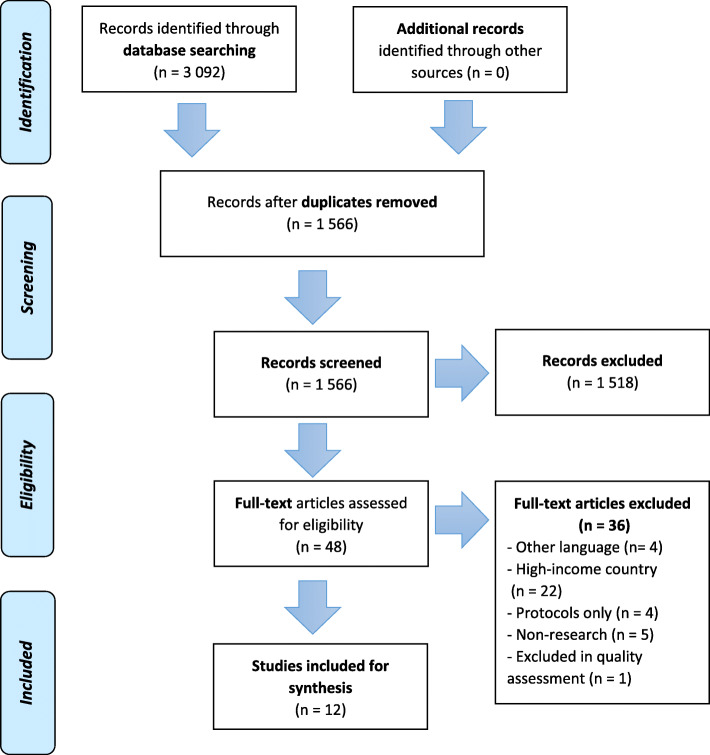
The PRISMA flowchart illustrating the screening and selection process

The PRISMA flowchart in Fig. 2 outlines the screening and selection process [24]. The electronic search strategy identified 3092 papers. After duplicate papers had been removed, and screening against titles and abstracts and full-text assessment of papers had been completed, 12 papers were included in synthesis.

### Characteristics of included papers

The papers included in the systematic review included six randomised controlled trials, one cohort study, two non-randomised trials, two single group, pre-post-test study and one systematic review. The participants of the various studies consisted of adults with T2D from various LMICs, such as Iran [30], India [31, 32], the Philippines [33], Mali [34], Malaysia [35], South Africa [36], Jamaica [37], Brazil [38], Guatemala [39], Cameroon [40] and a few other countries classified by the World Bank as LMICs [41]. The characteristics of the papers that were included are set out in Additional file 1. The two models of peer support that were identified in the systematic review and the subsequent emerging themes – recruitment, selection, training, mode, frequency and duration of peer intervention and supervision – are explained in Table 2.

**Table 2:** Themes emerging from the included papers from the two identified models of peer support: diabetic patients and community health workers

MODEL 1: DIABETIC PATIENTS		MODEL 2: COMMUNITY HEALTH WORKERS	
***THEME 1: RECRUITMENT***			
***SUB-THEME:***		***SUB-THEME:***	
**1. Responsibility**	**Recruited by healthcare professionals** at the clinic or by the research team [30, 31, 35]		
**2. Origin**	**From the local community** [30, 31, 33–35]	**1. Origin**	**CHWs from the local community from existing CHW infrastructure** [32, 36, 38, 39]
***THEME 2: SELECTION***			
***SUB-THEME:***		***SUB-THEME:***	
**1. Criteria:**	**Good glycaemic control** [30, 31, 35, 40]	**1. Criteria**	**Education levels** Some high school education [36, 38]; high school diploma [41]; primary education [41]
	**Leadership qualities** [30, 33–35, 40]		**Experience** Two years of training; healthcare or community experience; subjected to an entrance examination [41]
	**Achieve pass mark** [34]		**Achieve pass mark** [37, 39]
**THEME 3: TRAINING**			
***SUB-THEME:***		***SUB-THEME:***	
***1. Provision***	**Health-care specialists** [30, 31, 33, 35]	***1. Provision***	**Health-care specialists** [36, 39] **Trainers, trained by research members** [38]
***2. Duration***	**Two days** [31, 33, 35, 40] **Three days** [30] **Four days** [34]	***2. Duration***	**Initial four-days**, followed by two days [36] **Initial five days** followed by five days [39] **Four eight-hour days**, followed by four hours per month for six months [38] **10 eight-hour days** [32] **Initial six hours**, two hours follow-up [37]
***3. Content***	**Diabetes-specific information** [31, 33, 35, 40] **Communication skills** [30, 31, 34, 35, 40] **Tailoring information for the patient** [31, 33, 34, 40] **Effective individual and group management** [30, 40]	***3. Content***	**Diabetes-specific information** [32, 36–39] **Communication skills** [32, 36, 38, 39] **Tailoring information for the patient** [32, 36] **Behaviour change principles** [38, 39]
***4. Theoretical basis for training***	**Socio-constructivist theory** [34] **Social cognitive theory** [35]	***4. Theoretical basis for training***	**Motivational interviewing principles** [36, 38, 39]
**THEME 4: MODE OF PEER INTERVENTION**			
***SUB-THEME:***		***SUB-THEME:***	
**1. Group interventions**	[30, 33, 34, 40]	**1. Group interventions**	[36, 37, 39, 41]
**2. Individual interventions**	[31, 35]	**2. Individual interventions**	[32, 38]
**3. Additional strategy**	**Telephone** [30, 31, 35, 40]	**3. Additional strategy to group intervention**	**Individual face-to-face** [37, 39, 41]
**THEME 5: FREQUENCY OF PEER INTERVENTION**			
***SUB-THEME:***		***SUB-THEME:***	
**1. Weekly**	[30, 31, 33]	**1. Weekly**	[32, 39, 41]
**2. Monthly**	[30, 34, 35, 40]	**2. Monthly**	[36, 38, 41]
		**3. Three-monthly**	[37, 41]
**THEME 6: DURATION OF PEER INTERVENTION**			
***SUB-THEME:***		***SUB-THEME:***	
**1. Four weeks**	Follow-up at three and six months [33]	**1. 40 days**	[32]
**2. Three months**	[31] Follow-up at weeks 24 and 35 [35]	**2. Four months**	[36, 39, 41]
**3. Six months**	[30, 40]	**3. Six months**	[37, 38, 41]
**4. 12 months**	[34]	**4. 12 months**	[41]
**THEME 7: SUPERVISION OF PEERS**			
***SUB-THEME:***		***SUB-THEME:***	
**1. Audio-recording**	Group meetings were recorded and provided to research team for feedback [30]	**1. Evaluation of CHWs**	CHWs were evaluated by researcher at health centre and given feedback [36, 38]
**2. Telephone contact**	The research team contacted the peer supporters weekly [30]		
**3. Debriefing meetings**	Two fortnightly and two-monthly debriefing meetings [35]		
**4. Clinic visits**	Supervision at the monthly clinic visits, feedback was provided [35]		

### Models of peer support

Six papers reported on delivering peer support using diabetic patients [30, 31, 33–35, 40], and the other six papers delivered peer support using CHWs [32, 36–39, 41].

**THEME 1: RECRUITMENT**

**SUB-THEME: RESPONSIBILITY**

Diabetic patients were recommended as peer supporters by healthcare professionals at the clinic, or by the research team [30, 31, 35].

**SUB-THEME: ORIGIN**

Diabetic patients who were recruited as peer supporters were individuals from the community [30, 31, 33–35]. CHWs were recruited as peer supporters from the existing infrastructure of CHWs in the local community [32, 36, 38, 39].

**THEME 2: SELECTION**

**SUB-THEME: CRITERIA**

The selection of diabetic patients as peer supporters was guided by good glycaemic control [30, 31, 35, 40] and/or leadership qualities [30, 33–35, 40]. One paper evaluated diabetic patients after training; patients were selected only if they achieved a pass mark [34]. As part of the selection criteria of CHWs as peer supporters, three papers reported certain levels of education and/or experience [36, 38, 41], while two papers evaluated CHWs after training; they were selected only if they achieved a pass mark [37, 39].

**THEME 3: TRAINING**

**SUB-THEME: PROVISION**

Four papers [30, 31, 33, 35] reported that diabetic patients were trained as peer supporters by healthcare specialists; two other papers [34, 40] did not report who provided training to diabetic patients. In two papers [36, 39], training of CHWs as peer supporters was provided by healthcare specialists; another paper [38] used trainers who had been trained by the research team, and in the other three papers [32, 37, 41], it was unclear who had provided the training to the CHWs.

**SUB-THEME: DURATION**

The duration of the training of diabetic patients as peer supporters ranged from two to four days [30, 31, 33–35, 40]. The duration of the training of CHWs as peer supporters ranged from six hours to 10 eight-hour days [32, 36–39].

**SUB-THEME: CONTENT**

The content of the training of diabetic patients as peer supporters included diabetes-specific information, communication skills, effective individual and group management, and tailoring information for the patient [30, 31, 33–35, 40]. The content of the training of CHWs as peer supporters also included diabetes-specific information, communication skills, effective individual and group management and tailoring information for the patient, as well as behaviour change principles [32, 36–39].

**SUB-THEME: THEORY BASIS**

Only two papers [34, 35] reporting on diabetic patients as peer supporters used the social cognitive and socio-constructivist theories as the basis for training. Four papers [30, 31, 33, 40] did not report on a theory used as basis for training. Three papers [36, 38, 39] on CHWs as peer supporters used motivational interviewing principles as the theoretical basis for training, and three papers [32, 37, 41] did not report on using a theory as the basis for training.

**THEME 4: MODE OF PEER INTERVENTION**

**SUB-THEME: GROUP INTERVENTION**

For diabetic patients as peer supporters, four papers [30, 33, 34, 40] used group interventions, and for the CHWs as peer supporters, four papers [36, 37, 39, 41] used group interventions.

**SUB-THEME: INDIVIDUAL INTERVENTION**

In the papers with diabetic patients as peer supporters, two papers [31, 35] used individual interventions. In the papers with CHWs as peer supporters, two papers [32, 38] used individual interventions. Individual support was used additionally in three of the group intervention papers [37, 39, 41] in the CHW group.

**SUB-THEME: TELEPHONE INTERVENTION**

Four of the papers [30, 31, 35, 40] reporting on diabetic patients as peer supporters used telephone support as an additional peer support strategy.

**THEME 5: FREQUENCY OF PEER INTERVENTION**

The frequency of peer support by diabetic patients as peer supporters ranged from weekly to monthly [30, 31, 33–35, 40], and when CHWs served as peer supporters, the frequency ranged from weekly to every 3 months [32, 36–39, 41].

**THEME 6: DURATION OF PEER INTERVENTION**

The duration of the peer intervention when diabetic patients served as peer supporters ranged 4 weeks to 12 months [30, 31, 33–35, 40], and the duration ranged from 40 days to 12 months when CHWs were peer supporters [32, 36–39, 41].

**THEME 7: SUPERVISION OF PEERS**

Supervision of diabetic patients serving as peer supporters was reported in only two of the six papers [30, 35]; four papers did not report on supervision of diabetic patients serving as peer supporters [31, 33, 34, 40]. Supervision of CHWs serving as peer supporters was also reported in only two of the six papers [36, 38]; four papers did not report on supervision of CHWs as peer supporters [32, 37, 39, 41].

**SUB-THEME: AUDIO-RECORDING**

When diabetic patients were used as peer supporters, supervision was conducted by audio-recording group meetings, which was provided to the research team for feedback [30].

**SUB-THEME: TELEEPHONE CONTACT**

When diabetic patients served as peer supporters, the research team contacted the peer supporters weekly as a form of supervision [30].

**SUB-THEME: DEBRIEFING MEETING**

In studies using diabetic patients as peer supporters, two fortnightly and two-monthly debriefing meetings were held with peer supporters as a means of supervision [35].

**SUB-THEME: CLINIC VISITS**

When diabetic patients were peer supporters, supervision was performed by conducting monthly clinic visits and providing feedback [35].

**SUB-THEME: EVALUATION OF CHWS**

CHWs serving as peer supporters were evaluated by the researcher at the health centre, and feedback was provided [36, 38].

## Discussion

This study was based on the IMBP, which suggests that an individual will perform the intended behaviour if the necessary skills are present and if contextual factors allow the behaviour to be performed. Background factors cannot be ignored because they impact on attitudes, perceived norms and self-efficacy and eventually on the intention to perform the behaviour. We performed this systematic review because face-to-face peer support could serve as a strategy to develop self-management skills and minimise contextual constraints.

This review shows that diabetic patients and CHWs are commonly used models of face-to-face peer support, as a self-management strategy for T2D patients in LMICs. The review, furthermore, identified the following themes as fundamental to the planning and implementation of face-to-face peer support models: selection, recruitment, training, mode, frequency and duration of the peer intervention, and supervision of peer supporters.

The findings of the review were that the recruitment and selection criteria of diabetic patients as peer supporters focused on good glycaemic control and/or leadership qualities and/or a recommendation by a healthcare professional that a diabetic patient from the community would be a suitable peer supporter. For CHWs, recruitment took place from the existing infrastructure of CHWs within a community; criteria, such as educational levels and experience, were very poorly described. The training of peer supporters featured as an important component of the strategy, and healthcare specialists were commonly used to conduct training for both models. The duration of training ranged from 6 h to 10 days for the two models. Similar content was covered by the two models, and included diabetes-specific information, communication skills, effective individual and group management, tailoring information for the patient and behaviour change principles. Motivational interviewing was the most common theory basis used for the peer interventions. Face-to-face, either group or individual-based, peer support interventions were often supplemented by another method of peer support, and supervision of peer supporters was poorly described by studies using either model.

The studies in the reported review were associated with improvements in various clinical and behavioural outcomes. The improvement in outcomes included physical activity [35, 36, 38], mean diastolic blood pressure [31, 32, 36, 40], mean systolic blood pressure [32, 36], quality of diabetes care [30, 38], HbA1c [30–34, 37–41], consumption of fruit and vegetables [32, 38], medication adherence [31, 32, 38, 41], body mass index [31, 34, 41], cholesterol [31, 38, 40], waist circumference [34] and diabetes self-care behaviour [30, 40, 41].

The reported review shows that when diabetic patients are used as peer supporters, self-management by patients with T2D can be improved. A study conducted in a high-income country, supported the findings of the reported review although the context in high-income countries are different from LMICs. Thom et al. (2013) published a randomised controlled trial conducted in the United States of America to test whether clinic-based peer support would improve the glycaemic control of poorly controlled diabetic patients [42] . The study clearly describes utilising the electronic records of an institution to identify potential peers with an HbA1c of >8.5%, and who spoke either English or Spanish; other potential peer supporters were recommended by the clinic staff. Potential peer supporters attended 35 h of training over 8 weeks; the training was provided by healthcare specialists on specific content, but the study does not report the theoretical basis of training. Selection as a peer supporter was dependent on passing a written and oral examination. The peer intervention consisted of two or more group sessions over 6 months, supplemented by two telephone contacts per month. The study does not report on supervision. The study found a significant reduction in HbA1c in the peer support group.

Similarly, Baumann et al. (2015) conducted a study in Uganda to test the feasibility of a peer support intervention to improve diabetes self-care behaviours, glycaemic control, social support and emotional support and linkages to care [43]. The clinic staff recruited participants at the clinic and via radio. Peer supporters had to have type 2 diabetes, speak and read English, be willing to receive training in communication skills, and agree to weekly contacts with patients. Patients had to agree to weekly contact with peer supporters. Specialists in diabetes care delivered diabetes training to the peer supporters for 5 h and an additional hour on communication skills and the daily management of diabetes. The training was provided in English on 1 day, and on another day, diabetes training was delivered to the patients for 5 h in English and the local language. Peer supporters and patients met on the patient training day. They were matched on age and gender and agreed to contact each other weekly either by personal contact or by telephone during the four-month period. All participants were provided with mobile phones with a prepaid network. Participants received a logbook to record all contact with peers. The results of the study demonstrated improvements in self-reported eating behaviour, HbA1c, and diastolic blood pressure. The study also showed that 93% (*n* = 40) of contact was by cell phone and 60% (*n* = 28) by personal contact.

The reported review also shows that when CHWs are used as peer supporters, self-management by patients with T2D can be improved. Spencer et al. (2011) conducted a randomised controlled trial in the United States and tested the effectiveness of a CHW intervention amongst African American and Latino adults with T2D [44]. The study reports that ethnically matched CHWs from each community were assigned to the two communities. They received 80 h of training by the research team; the training combined empowerment approaches and social cognitive theory principles. Content covered was diabetes-specific content and behaviour change principles. The peer intervention consisted of eleven two-hour group sessions held every 2 weeks by the CHWs, and two home visits per month, combined with telephone contact every 2 weeks. The research team supervised one group session per CHW to evaluate fidelity. The results of the study showed a mean HbA1c improvement in the intervention group, as well as a greater self-reported understanding of diabetes compared to the control group.

Similarly, De Souza et al. (2017) conducted a randomised control trial in Brazil to assess the effect of a diabetes education program delivered to community health workers in improving the metabolic control of patients with type 2 diabetes mellitus [45]. The selection and recruitment of CHWs were not reported in the study as the CHWs were part of a primary care unit in a ‘Family Health Strategy Model’ serving a specific area of the population. Four CHWs was assigned to the intervention group that received structured weekly diabetes training for about 60 min for 1 month by the researcher and four CHWs to the control group, that received health education sessions on diabetes, asthma, tuberculosis, and contraception of about 60 min for 1 month by the researcher. Knowledge of participants and CHWs were tested before the intervention and after the intervention. CHWs in both groups visited the participants once a month and delivered information as received during training; namely, the intervention group provided diabetes training, and the control group offered general training. Three months after the training, baseline measurements were repeated on participants and displayed in a significant reduction in HbA1c in both intervention and control groups. High-density lipoprotein cholesterol levels and stress-related scores also reduced in both groups.

Opposing perspectives regarding the effectiveness of peer support in diabetes self-management also exist. A systematic review, on the effect of peer support on diabetes outcomes in adults, included 25 studies from high-income countries. The study also found that evidence was too inconsistent to support the endorsement of diabetes peer support [14]. Similarly, Werfalli et al. (2020) conducted a recent systematic review on the effectiveness of peer and CHW-led diabetes self-management programs in LMICs in diabetes care [46]. After synthesising eleven papers, the results were associated with inconsistency in terms of improvement in clinical, behavioural, and psychological outcomes. Based on the findings of the reported review and other prior studies, it can be concluded that the identified face-to-face peer support models may be used as a self-management strategy for patients with T2D in LMICs. Peer support is complex and the use of these face-to-face peer support models may minimise some of cultural, social and contextual constraints experienced by patients with T2D. The generalisability of the models will depend on available and existing resources within the context, and it is imperative that this is taken into consideration.

### Limitations

A limitation of the review is that new studies related to the review question may have been generated since the cut-off date of the review. However, an update of the systematic review will be performed as soon as possible. Another limitation of the study is that assessment from the included studies was limited to published data only.

## Conclusion

The results of this review suggest that face-to-face peer support models, either individual or group-based, can be used as a self-management strategy for patients with T2D in LMICs. The results suggest that diabetic patients and/or CHWs can be used to provide face-to-face peer support to patients with T2D in LMICs. The results provide further evidence that suggests that effective recruitment, selection, training and supervision of peer supporters are essential in the context of peer support models. The results of the review will be valuable for developing countries with resource-scarce settings.

### Implications for further research

Based on the results of the review, these findings inform researchers on the acceptability of face-to-face peer support models, either individual or group-based, in LMICs. Furthermore, the results can assist in the development and planning of face-to-face peer support interventions that aim to improve the self-management of patients with T2D in LMICs and the importance of considering contextual factors.

## Supplementary Information

**Additional file 1.** Characteristics of studies included in the study.

## Data Availability

Not applicable. All data has been provided within the manuscript and additional supporting files.

## Abbreviations

T2D: Type 2 diabetes
LMICs: Low and middle-income countries
CHWs: Community health workers
HbA1c: Glycosylated haemoglobin
GNI: Gross national income
IMBP: Integrated model of behaviour prediction
PRISMA: Preferred reporting items for systematic reviews and meta-analyses
CASP: Critical appraisal skills programme
