# Supplementary material for: Self-management in face-to-face peer support for adults with type 2 diabetes living in low- or middle-income countries: a systematic review
Source: BMC Public Health. 2020 Nov 30;20:1834. doi: 10.1186/s12889-020-09954-1 (PMC7706053; doi:10.1186/s12889-020-09954-1)
Supplement: Supplementary file 1 — Additional file 1. Characteristics of studies included in the study. [file 12889_2020_9954_MOESM1_ESM.pdf]

**Additional file 1: Characteristics of studies included in the study**

| STUDY AUTHOR, YEAR, COUNTRY AND DESIGN | AIM OF STUDY AND OUTCOMES MEASURED | SETTING AND STUDY POPULATION | QUESTIONS RELATED TO THE REVIEW QUESTION | | | | | | |
|---|---|---|---|---|---|---|---|---|---|
| | | | MODEL OF PEER SUPPORT | PEER SELECTION AND RECRUIT-MENT | PEER TRAINING AND THEORY BASIS | PEER INTERVENTION AND DURATION | PEER SUPPORT SUPERVISION | FINDINGS OF THE STUDY | QUALITY ASSESS-MENT |
| 1.<br>Peimani et al. (2018) [30]<br><br>Iran<br><br>Randomised control trial | **Aim:** To assess the effectiveness of a peer support intervention<br><br>**Outcomes measured:**<br>1) improvement in self-care activities<br>2) reported self-efficacy<br>3) quality of life<br>4) clinical outcomes: glycosylated haemoglobin (HbA1c) and body mass index | **Setting:** A diabetic speciality clinic<br><br>**Study population**: 200 T2DM patients aged 25-75; in intervention n=100/ 100 and control n=100/100 pre- and post-intervention respectively | Delivered by diabetic patient | **-** 10 diabetic patients nominated by physicians and diabetes educators<br>- based on specific criteria, such as knowledge on diabetes control, interpersonal skills, self-motivation and problem-solving skills | - attended three-day course<br>- structured, buzz-group interactive course<br>- conducted by research team<br>- content of training included developing facilitation, active listening and problem solving skills, providing emotional support, facilitating behaviour change, building boundaries and dealing with difficult situations<br><br>- theory basis for training not reported | - monthly face-to-face group-based session<br>- combined with weekly telephone support<br>- each peer assigned to 10 patients<br>- intervention duration six months | - group meetings were recorded<br>- recordings provided to research team<br>- peer supporters had weekly telephone contact with research team to provide support | 1**) Increase in mean self-care activities in peer support** (P<0.001)<br>**2) Mean self-efficacy** reduced in peer support (P<0.001)<br>3) **Increase in mean quality of life in peer support group** (P<0.001)<br>4) Decrease in mean **HbA1c value** in peer support group (P=0.045) | Good quality rating |
| 2.<br>Sreedevi et al. (2017) [31]<br>India | **Aim:** To study the feasibility and effect of yoga and peer support on glycaemic outcomes, | **Setting:** A rural area at a health-training centre | Delivered by diabetic patient | **-** three diabetics identified and recruited from community<br>- based on | - two-day training<br>- by specialists<br>- content of training included developing communication skills, diabetes-specific | **-** weekly face-to-face individual session<br>- a follow-up telephone call the same week | - not reported | 1) **FPG** decreased in yoga group (CI 34.5 – 44) compared to peer (CI 37.6 – 46.1) and control group (CI-31.6 – 28) | Good quality rating |

| | | | | | | | | | |
|---|---|---|---|---|---|---|---|---|---|
| An open parallel three-armed randomised control trial (feasibility study) | pharmacological adherence and anthropometric measures<br><br>**Outcomes measured**:<br>1) fasting plasma glucose (FPG)<br>2) HbA1c<br>3) quality of life<br>4) pharmacological adherence<br>5) body mass index<br>6) waist-hip ratio<br>7) blood pressure<br>8) total cholesterol | **Study population:** 124 T2DM women aged 30–65; randomised into yoga 41/32; peer intervention 42/32, and control groups 41/35 pre- and post-intervention respectively | | specific criteria, such as having T2DM ≥1 year; adherent to treatment; committed and capacity for training | information on diet, medication, exercise and dealing with the illness<br><br>- theory basis for training not reported | - one peer assigned to 13-14 patients<br>- intervention duration three months | | 2) Yoga group only decreased HbA1c (CI -0.85 – 0.34)<br>3) Quality of life not reported on<br>4) **Adherence** score increased in all arms<br>5**) Body mass index** increased in all arms<br>6) A mean fall **in waist-hip ratio** in peer and control groups at .04 and 0.03 respectively<br>7) Yoga group decreased in **diastolic blood pressure** (CI -0.64-0.77)<br>8) Total **cholesterol** levels in peer group decreased by 5 mg% (95% CI - 15, 5.1) | |
| 3. Paz-Pacheco et al. (2017) [33] Philippines<br><br>Prospective education-intervention trial | **Aim:** To assess the effectiveness of community-based diabetes self-management education (DSME) in a rural agricultural town<br><br>**Outcomes measured:**<br>1) body mass index<br>2) waist-hip ratio<br>3) blood pressure | **Setting:** Rural town<br><br>**Study population**: 155 T2DM patients from 19 randomised villages; 85/72 in DSME group and 70/52 in control group | - Delivered by diabetic patient | - 14 diabetic patients recruited from participants<br>- No criteria applied, only willingness to be trained<br><br>- after training, they had to demonstrate in return | - two-day workshop<br>**-** conducted by endocrinologists<br>- content of peer training included overview of diabetes mellitus, diabetes and exercise, diabetes and diet, pharmacological treatments and complications of diabetes | - weekly face-to-face group-based session<br>- six to 15 participants per group<br>- intervention duration four weeks only<br>- data repeated at three and six months. | Supervision - not reported | 1) **No body mass index** changes<br>2) **Waist-hip ratio** for women higher in DSME group than for usual care at third month (*p* = 0.02)<br>3) **Diastolic blood pressure**, no significant changes<br>4) DSME group had lower median **HbA1C levels** at the third | Moderate quality rating |

| | | | | | | | | | |
|---|---|---|---|---|---|---|---|---|---|
| | 4) HbA1c<br>5) FBG<br>6) cholesterol<br>7) exercise<br>8) foot examinations<br>9) cigarette smoking, illicit drug use and alcohol consumption<br>10) medication adherence | pre- and post-intervention respectively | | | - theory basis for training not reported | | | (P=0.03) and sixth months (P=0.01)<br>**5) FBG levels** similar for both groups at all points<br>**6) Total cholesterol levels**, decrease in third month in DSME group compared with usual care group (P=0.0002), as well as on the sixth month (P=0.0002)<br>7) R**egular exercise** in DSME group was comparable with usual care group by third and sixth months, at baseline (34.94 versus 51.43%, P=0.040)<br>8) In both groups, **foot examination** increased by sixth month compared to baseline (from 24.10 to 75.81% in DSME group, and from 41.43 to 85.11% in usual care group).<br>9) Groups were similar regarding **smoking cigarettes, consuming alcohol and using illicit drugs**<br>**10) Medication compliance** similar in both groups | |

| | | | | | | | | | |
|---|---|---|---|---|---|---|---|---|---|
| 4.<br>Debussche et al. (2018) [34]<br>Mali<br><br>Open-label randomised controlled trial | **Aim:** To evaluate the effectiveness of peer-led self-management education in improving blood glucose control in patients with T2DM<br><br>**Outcomes measured:**<br>1) HbA1c<br>2) weight and body mass index<br>3) waist circumference<br>4) systolic and diastolic blood pressure<br>5) anti-diabetic treatment<br>6) knowledge score<br>7) dietary practices | **Setting:** Two secondary health centres<br><br>**Study population:** 151 T2DM patients aged 30-80; in intervention group 76/70 and control group 75/70 pre- and post-intervention respectively | Delivered by diabetic patient | - 10 diabetic patients recruited from local diabetic association<br>- based on having diabetes, living in the area, regular checks with a physician, volunteering for educational sessions, and being fluent in both local languages<br>- patients evaluated before selection, only five were chosen for the project | - initial four-day training,<br>- not stated by whom<br>- content of peer training included focus on cardiovascular risk management, food intake, exercise, blood glucose and insulin management, taking into account individual, social and cultural context<br><br>- training based an empowerment-based approach derived from socio-constructivist theory | **-** face-to-face group sessions<br>- three courses over one year<br>- four themes per course, offered over three months (months 1-3, 7-9, and 10-12)<br>- content tailored for patients' literacy level and Mali culture<br>- four to 10 participants per session | Supervision not reported | 1) **HbA1c levels more favourable** in intervention (P=0.006)<br>2) **Body mass index more** favourable in intervention (P=0.0005)<br>3) **Waist circumference** more favourable in intervention (P=0.0003)<br>4) Changes in **systolic pressure** between intervention and control group was P=0.003. Changes in diastolic pressure between intervention and control group was P=0.36<br>5) Proportion of patients **receiving insulin** decreased in the intervention group (P=0.0003)<br>6) Changes in **knowledge levels** between intervention and control group was P=0.17<br>7) No positive change in **diet diversity score** | Good quality rating |

| | | | | | | | | | |
|---|---|---|---|---|---|---|---|---|---|
| 5.<br>Sazlina et al. (2015) [35,52]<br><br>Malaysia<br><br>A three-arm randomised controlled trial | **Aim:** To evaluate the effectiveness of personalised feedback (PF) about physical activity patterns alone or in combination with peer support (PS), in addition to usual diabetes care in improving physical activity levels in sedentary older Malays with T2DM<br><br>**Outcomes measured:**<br>1) physical activity level<br>2) subjective measure of physical activity<br>3) HbA1c; BP; body composition, i.e. weight; body mass, waist circum-ference, body fat percentage and lipid profiles<br>4) functional status (cardiorespiratory fitness and balance)<br>5) quality of life<br>6) psychosocial wellbeing | **Setting:** A primary healthcare clinic<br><br>**Study population:** 69 T2DM patients ≥60 years of age; randomised in PF group 23/19; PS group 23/17 and control group 23/16 pre- and post-intervention respectively | Delivered by diabetic patients | - clinic staff personally contacted potential peers telephonically<br>- notices displayed at clinic<br>- specific criteria applied: must volunteer; have T2DM ≥5 years; lived in same community; engaged in regular physical activity; HbA1c of 8%; owns a mobile phone; willing to attend training | - 2-day training<br>- not stated by whom<br>- content of training included diabetes self-management, physical activity, stress management and communication<br><br>- the training intervention incorporated constructs of social cognitive theory | - three face-to-face individual sessions and<br>- three telephone sessions<br>- intervention duration 12 weeks<br>- follow-up at 24 and 36 weeks | -two fortnightly and<br>- two-monthly debriefing meetings<br>- held over the 12 weeks,<br>- to facilitate and support the peer mentor<br>- ongoing supervision at monthly clinic visits with their peers, where feedback was provided to peer mentors on their performance and possible points of improvement | 1) PS group showed greater daily **pedometer readings** than PF group and control group (P=0.001)<br>2) PS group had greater improvement in weekly duration (P<0.001) and frequency (P<0.001) of **physical activity**<br>3) No changes in HbA1c, weight, body mass index and waist circumference. Body fat reduced more in PS than PF and control group (P=0.004)<br>4-6) PS showed greater improvement in the 6-min walk test (P<0.001), physical activity scale for elderly (P=0.003) and social support from friends (P=0.032) than PF and control groups | Good quality rating |

| | | | | | | | | | |
|---|---|---|---|---|---|---|---|---|---|
| 6.<br>Mash et al. (2014)<br>[36,53]<br>Cape Town<br><br>Pragmatic cluster randomised controlled trial | **Aim:** To evaluate the effectiveness of group diabetes education in under-served communities in South Africa<br><br>**Outcomes measured:**<br>1) improved self-care activities<br>2) 1% HbA1c reduction<br>3) 5% weight loss<br>4) improved locus of control<br>5) self-efficacy<br>6) mean weight loss<br>7) blood pressure<br>8) waist circumference<br>9) mean total cholesterol<br>10) quality of life | **Setting:** 34 Public sector community health centres<br><br>**Study population:**<br>1 570 T2DM patients recruited, 710/391 in intervention arm from 17 health centres, 860/475 in control arm from 17 health centres in pre- and post-intervention respectively | Delivered by CHWs | - CHWs with secondary school education were recruited from district health service | - initially trained in four-day workshop<br>- training conducted by family physician and nurse diabetic educator<br>- a further two-day workshop two months later<br>**-** content included understanding diabetes**,** **l**iving a healthy lifestyle, understanding the medication and preventing complications<br>- education and activities adapted to local context<br><br>- training based on motivational interviewing guidelines | - monthly face-to-face group sessions<br>**-** 10-15 patients per group<br>**-** intervention duration six months<br>- follow-up 12 months | **-** health promoters were evaluated by the researcher at each health centre at least twice and gave feedback to the health promoters after the sessions | 1) No differences in **self-care activities,**<br>2) **HbA1c level** by 1% (CI 0.64–1.73) or<br>3) in achievement of **5% weight loss** (CI 0.50–1.24) 4) No significant differences in **locus of control,**<br>**5) self-efficacy,**<br>**6) weight,**<br>**7)** s**ignificant decrease in mean systolic blood pressure**(-4.65 mmHg, 95% CI 9.18 to -0.12; P=0.04) **and diastolic blood pressure** (-3.30 mmHg, 95% CI -5.35 to -1.26; P=0.002) in intervention group<br>8) No significant differences in waist circumference,<br>9) total cholesterol or<br>10) quality of life | Good quality rating |
| 7.<br>Less et al. (2010)<br>[37]<br>Jamaica<br><br>Prospective cohort study | **Aim:** To evaluate the effectiveness of lay diabetes facilitators to increase knowledge and improve control in persons with diabetes | **Setting:** 16 health centres<br><br>**Study population:**<br>318 T2DM patients aged 25–75 years; recruited from | Delivered by CHWs | - not reported<br><br>- after training, 24 achieved >90% and qualified for this study | - 42 CHWs were trained for six hours<br>- not stated by whom<br>**-** content included basic knowledge on diabetes, management, complications and self-monitoring | **-** three-monthly face-to-face group sessions **and**<br>**-** three-monthly home-visits<br>- 10 -12 participants per group | Not reported | 1) Mean HbA1c for both groups similar at baseline (7.9% versus 8.0%; P>0.58). At 6 months, intervention group reduced HbA1c by 0.6% while the comparison group had an | Good quality rating |

| | | | | | | | | | |
|---|---|---|---|---|---|---|---|---|---|
| | **Outcomes measured:**<br>1) Improvement in HbA1c | 16 health centres;<br>8 centres intervention group, 8 centres control group,<br>n=159 intervention;<br>n=159 control group | | | - CHWs retrained in a two-hour diabetes session at baseline and six months<br><br>- theory basis for training not reported | - intervention duration six months<br><br>- CHW used three patient self-monitoring forms**:** personal eating tracker; physical activity log; and blood glucose monitoring form<br>**-** CHW reviewed these forms at each session | | increase of 0.6% (P<0.001) | |
| 8.<br>Dasappa et al. (2016) [32]<br>India<br><br>Non-randomised controlled trial | **Aim:** Assessing the effectiveness of yoga, pranayama, and sudarshan kriya in community-based management of diabetes mellitus<br><br>**Outcomes measured:**<br>1) Hb1Ac<br>2) systolic and diastolic blood pressure<br>3) adherence to medication<br>4) changes in lifestyle | **Setting:** Four urban slums<br><br>**Study population:**<br>109 diabetes patients from four slums, mean age of 52, 94%;<br>52/40 in intervention (agreed to learn and practice yoga);<br>remaining 57 assigned to non-intervention group | Delivered by CHWs | - no criteria reported<br>- four local women recruited from same area to implement the project | **-** CHWs had 81 hours of training<br>- content included 56 hours on leadership qualities and other soft skills<br>- 10 hours basics of diabetes and hypertension<br>- 15 hours on measuring blood sugar, blood pressure and weight<br>- not reported who conducted training<br><br>- theory basis for training not reported | - Weekly face-to-face individual intervention<br>- They filled in follow-up information sheets that included glucose random blood sugar, blood pressure, weight, physical activity, dietary practices, adherence to medications by pill count, smoking, and alcohol consumption | Not reported | 1**) Mean HbA1c** decreased in intervention and non-intervention group, insignificantly, due to short duration of intervention<br>2) Significant change in **systolic and diastolic blood pressure** ≤140/90 ($x2 = 10.635$, P<0.005) between two groups<br>3) **adherence to metformin** (P<0.005), and other medication improved (P<0.005)<br>4) **vegetable consumption** improved (P<0.005); | Good quality rating |

| | | | | | | | | | |
|---|---|---|---|---|---|---|---|---|---|
| | | | | | | - CHWs made a total of six visits during the study<br>- The intervention duration was **40 days** | | **fruit consumption** improved (P<0.005);<br>**salty food consumption** reduced (P<0.005);<br>**bakery food consumption** reduced (P<0.005) and<br>**fried food consumption** reduced (P<0.005) | |
| 9.<br>do Valle Nascimento, et al. (2017) [38]<br>Brazil<br><br>Single group, pre-post study | **Aim:** To examine the feasibility, acceptability, and outcomes of training community health agents in motivational interviewing-based counselling for patients with poorly controlled diabetes in a primary care centre in São Paulo, Brazil<br><br>**Outcomes measured:**<br>1) participants' report on quality of diabetes care, measured by the Patient Assessment of Chronic Illness Care | **Setting:** A primary care health unit<br><br>**Study population:**<br>57 T2DM patients aged <75 years.<br>52 patients completed the study | Delivered by CHWs | - CHWs recruited from residents of the communities<br><br>- no formal training required besides some secondary education | **-** 19 CHWs participated in 32 hours of initial training<br>- led by Brazilian trainers trained by research members<br>- CHWs **had four hours a** month additional **booster training and support** over six-month intervention period.<br>**- content included**<br>1) identify personal **diabetes self-management goals**<br>2) **formulate specific short-term** action plans to reach goals<br><br>- motivational interviewing-based theory | - monthly face-to-face individual home visits<br>- six-month intervention duration | - CHWs were evaluated at home visits by the research team | 1) **PACIC improved** (P <.001)<br>2) Significant increase in consumption **of fruits and vegetables** (P<.001),<br>3) in **physical activity** (P=.001), and<br>4) in **diabetes medication adherence** (P=.002).<br>5) insignificant decreases in **consumption of fat foods (P=.402) or sweets** (P=.436).<br>6) six-month **A1c levels 0.34% points** lower than at baseline (P=.08)<br>7) improved mean LDL (P=.005) and | Good quality rating |

| | | | | | | | | | |
|---|---|---|---|---|---|---|---|---|---|
| | (PACIC)<br>2) consumption of fruit and vegetables<br>3) physical activity<br>4) diabetic medication adherence<br>5) consumption of fat and sweets<br>6) A1C<br>7) cholesterol levels<br>8) triglyceride levels | | | | | | | 8) improved **triglyceride levels** (P=.002) | |
| 10. Micikas et al. (2012) [39] Guatemala<br><br>A single-group, pre-test post-test design | **Aim:** To determine if a structured, community-led diabetes self-management intervention could be implemented successfully and whether the intervention could improve selected health outcomes for diabetic patients<br><br>**Outcomes measured:**<br>1) changes in HgA1c<br>2) body mass index<br>3) health beliefs and practices of | **Setting:** Two rural primary care clinics<br><br>**Study population:** 52/52 T2DM patients ≥18 years participated in pre- and post-test surveys | Delivered by CHWs | -not reported<br><br>- after training and evaluation, the team **selected 8 CHWs** for the programme | - 21 CHWs participated in **one week** of **daily "train-the-trainer" sessions**<br>- content focused on the diabetes disease process and principles of management<br>- the selected CHWs had an **additional week of training**.<br><br>- motivational interviewing and health behaviour-based theory | - weekly face-to-face group meeting<br>-15-20 patients per group<br>- CHWs also conducted weekly home visits and<br>- pre-consultations at clinic<br>- intervention duration four months | Not reported | 1) the mean HgA1c for sample decreased to 8.9%, a statistically significant decrease (P=.001)<br>2) no significant change in mean body mass index<br>3) results from health beliefs and practices survey were mainly non-significant | Moderate quality rating |

| | | | | | | | | | |
|---|---|---|---|---|---|---|---|---|---|
| | participating diabetic patients | | | | | | | | |
| 11.<br>Assah et al. (2015)<br>[40]<br>Cameroon<br><br>Non-randomised control trial | **Aim:** To examine the effectiveness of a community-based multi-level peer support intervention<br><br>**Outcomes measured:**<br>1) Improved glycaemic levels<br>2) blood pressure<br>3) lipids in patients with T2DM<br>4) diabetes self-care behaviour | **Setting:** National Obesity Centre, at a hospital<br><br>**Study population:** 100/96 T2DM patients in intervention arm and 100/96 were recruited into the control arm in pre- and post-intervention respectively | Delivered by diabetic patient | Diabetic patients recruited based on better glycaemic control, more compliant with clinic visits and experiential knowledge on diabetes – they had to volunteer | - two- day **training**<br>- content included building and reinforcing **knowledge on diabetes**, training on **communication skills**, effective **group and face-to-face meetings**, and use of **personal history in peer support**.<br><br>- theory base for training not reported | **- m**onthly **face-to-face group meetings**<br>**- 8-10 participants per group, 10 groups**<br>- also **monthly individual sessions** and **phone calls**<br>**-** intervention **duration six months** | Not reported | 1) significant reduction in **HbA1c** in intervention group compared with controls (P <0.001)<br>2) significant reductions in **diastolic pressure** (P<0.001)<br>3) Significant reductions **in cholesterol** (P<0.001), HDL (P<0.001), BMI (P<0.001)<br>4) diabetes self-care behaviours (general diet, exercise, blood glucose testing and foot score) improved significantly (P<0.001) | Good quality rating |
| 12.<br>Alaofè et al. (2017)<br>[41]<br>Developing countries<br><br>Systematic review | **Aim:** To critically appraise evidence regarding the effectiveness of CHW interventions for prevention and management of T2DM in LMICs<br><br>**Outcomes measured:**<br>Outcomes that | **Setting:** LMICs<br><br>**Study population:**<br>**n = 6 297** studies after filtering: title and abstract, inclusion and exclusion criteria and | Delivered by CHWs | - only three of nine studies reported on selection criteria, ranging from primary education, high school education, leadership qualities, | - only three studies reported training duration, ranging between six hours, four weeks and 14 weeks respectively<br>- five studies reported training content: diabetes lifestyle modifications, medication awareness | - seven of nine studies reported face-to-face interventions<br>- two studies did not report the nature of the intervention<br>– three studies reported conducting either group or | Not reported in any study | - 10 studies were included (four pre- and post-studies, two randomised controlled trials, two cohort studies, one cross-sectional study, and one case-control study)<br>-positive outcomes reported in seven of 10 studies. These outcomes included | Moderate quality rating |

| | | | | | | | | | |
|---|---|---|---|---|---|---|---|---|---|
| | might result from CHW-led T2DM care or prevention interventions | full text analysis.<br>**n = 10 articles** included: 2 prevention and 8 management studies<br><br>**Studies included**<br>Randomised controlled, cross-sectional, cohort, controlled before-and-after and case-control studies published from inception to May 31, 2017 | | bilingualism, community experience, an entrance examination and years of training | and prevention of complications<br>- none of the studies reported who provided the training<br><br>- theory base for training not reported in any study | individual sessions<br>- four studies reported a combination of group and individual peer support intervention<br>- intervention period ranged from three to six months, with varying follow-up periods | | increased knowledge of T2DM symptoms and prevention measures, increased adoption of treatment seeking and prevention measures, increased medication adherence, and improved fasting blood sugar, glycated haemoglobin, and body mass index. Three studies showed no significant outcomes | |
